# AGE-RELATED DIFFERENCES IN EVERYDAY SOLITUDE SEEKING WHEN TIME WITH OTHERS FEELS LESS DESIRABLE

**DOI:** 10.1093/geroni/igae098.3498

**Published:** 2024-12-31

**Authors:** Yoonseok Choi, Hansol Kim, Christiane Hoppmann

**Affiliations:** University of British Columbia, Vancouver, British Columbia, Canada; Sogang University, Seoul, Republic of Korea; The University of British Columbia, Vancouver, British Columbia, Canada

## Abstract

People spend a significant amount of time by themselves. This project seeks to investigate one potential motive underlying solitude seeking in daily life, namely, to disengage from undesirable social interactions. Social relationships and interactions often involve obligations to others, particularly so in collectivistic cultures; meeting obligations can be taxing when social demands exceed one’s capacity. Seeking solitude may be a way to reduce obligation-related social strains. Additionally, there may be age-related differences in motive strength considering age-related differences in social relationship structures, with higher obligatory demands placed on younger adults (e.g., work, childcare). To examine these ideas, we used 610 repeated daily life assessments (2 times per day for 10 days) collected from an adult lifespan sample of 51 Koreans (Age range: 22-71 years; 65% female, 98% some postsecondary education). Everyday assessments of preference for solitude were predicted by the extent to which previous-day social interactions were deemed voluntary and by their interaction with age. Results based on multilevel modeling showed that while previous-day reductions in voluntary social interaction ratings were not significantly associated with next-day increases in preference for solitude, those who reported generally lower levels of voluntary social interactions showed generally higher preferences for solitude (OR = 0.70, p =.001). Age moderation was significant in the predicted direction with younger age being associated with higher preference for solitude from lower voluntary social interactions (OR = 1.02, p =.002). These results highlight a possibly restorative function of solitude that plays out differently across the adult lifespan.

